# Comparison of the worst-case scenarios between training and competition weeks for each playing position in an elite football season

**DOI:** 10.5114/biolsport.2025.148538

**Published:** 2025-04-28

**Authors:** Adrián Díez, A. Vanessa Bataller-Cervero, Elena Mainer-Pardos, Alberto Roso-Moliner, José Luis Arjol-Serrano, Demetrio Lozano

**Affiliations:** 1 Universidad San Jorge, Autovía A23 km 299, Villanueva de Gállego, 50830 Zaragoza, Spain

**Keywords:** Soccer, Monitoring, Performance, Acceleration Deceleration

## Abstract

The aim of this study was to examine the worst-case scenarios (WCS) produced during the week in training sessions and compare them with the physical demands of the matches. A total of 194 training sessions and 42 matches were analysed during the 2020/2021 season in the Second Spanish Football League. Data were collected using Global Positioning System devices. Players were grouped according to their playing positions into central defenders, wide players, midfielders (MID), and forwards. The variables analysed were distance, HSR distance, sprint distance, high acceleration, high deceleration, and high metabolic load distance. The most demanding passages were analysed in 1-minute periods using the rolling average method. The most significant differences were found in the HSR distance (p < 0.001; ƞp^2^ = 0.854), sprint distance (p < 0.001; ƞp^2^ = 0.882) and high metabolic load distance (p < 0.001; ƞp^2^ = 0.899) variables on the day furthest from the match day. No significant differences were found in the data analysed for MID in any training session of the week compared with the match, nor in the variables of high acceleration and high deceleration. Knowledge about the worst-case scenario during a competitive microcycle can help understand the physical level and individual requirements that our players need to perform at a high level on the match day, as well as preventing possible injuries or fatigue.

## INTRODUCTION

Football, in terms of conditional capacities, is considered an explosive team sport characterised by the repetition of intermittent periods of anaerobic efforts, interspersed with long recovery periods [1]. These intermittent periods of anaerobic effort include repeated sprints, rapid accelerations and decelerations, changes in direction, jumps, throws, and post-jump activities [2]. All these demands are unique to each context and are unpredictable in terms of both intensity and duration [2].

In recent years, technological advances have enabled the development of tools that facilitate sports activities [3]. In sports, these tools allow for control of the athletes’ workload, which can be divided into two main groups: 1) technologies for controlling the athlete’s external workload and 2) technologies for controlling the athlete’s internal workload [4–9]. External workload is defined as the objective measures of activity performed by the athlete during training and competition, for instance, speed, acceleration, strength and power [10]. The measures that are mainly found in studies are the demands of total distance, high sprint running distance, sprint distance [11], the number of sprints, the number of accelerations and decelerations [12], the acceleration and deceleration distance, the metabolic power, high metabolic load distance (HMLD), player load and maximum speed [13]. Internal workload is defined as the psychophysiological response of athletes with physical exercise. Internal workload includes psychological measures such as subjective perception of effort (rate of perceived exertion – RPE), and physiological measures, such as heart rate, blood lactate or oxygen consumption [9]. Although they may be closely related, external workloads are assessed independently from internal workloads [10]. Due to workload control technologies, training can be individualised and optimised, reducing the risk of potential injuries [14].

One of the most commonly used technologies for monitoring the external workload in team sports is electronic performance tracking devices (EPTS) [15]. These are further divided into 3 main groups: 1) Global Positioning System (GPS) devices, 2) local positioning systems (LPS), and 3) video tracking systems (VTS) [14]. These technologies can also currently be combined with micro-electromechanical measurement systems (MEMS), making use of inertial sensors [16], which usually consist of accelerometers that make the devices much more beneficial and can provide us with many more measures for exercise analysis. These devices offer metrics that complement tracking systems, such as accelerations, decelerations, turns, changes of direction, jumps, impacts, or player load (defined as the accumulation of movement in the three axes of motion) [17].

For several years in football, physical demands have been quantified during training and matches to organise training based on the demands provided by data extracted from competitions [11]. These demands can be analysed weekly, monthly, or across different seasonal phases [18]. Thus, it is appropriate to quantify the workload because continuous workload following is important to quantify aspects such as individual responses to training, accumulative fatigue, preparation, and recovery status [6].

The physical demands of football players during matches are widely recognised [2]. However, these demands may vary depending on the player’s position on the field or the playing systems used by coaches during the season [19]. These findings demonstrate the significance of individualising both by athlete and position [2].

To quantify workload and programme weekly training sessions in an individualised and optimised manner, coaching staff have traditionally used average data derived from physical demands obtained in matches of chosen conditional variables. Therefore, it is important to consider the maximum demands when planning training sessions. However, using these averages underestimates the maximum demands produced in competition [20]. To address this underestimation, another method was employed, which considers the moments during the match when the highest physical demands of each variable occur. This method is known as the worst-case scenarios (WCS) or most demanding passages (MDP) [16]. The analysis of these WCS initially involved using fixed-length periods, which divide the total match into fixed periods from the beginning to the end of the match, for instance, periods of 1 minute (0.00’’-0.59’’, 1.00’’-1.59’’, continuing in this manner until the end of the match). However, currently, the rolling average is used, which involves fixing the periods when the WCS reaches its peak intensity. For example, the peak distance occurs in the period between 15.25’’ and 16.25’’, the peak sprint occurs between 24.49’’ and 25.49’’, and similar patterns continue [21].

It is common to think that identifying scenarios of maximum demand and replicating them during the week could aid in a player’s recovery and reduce the risk of injury [22]. However, there is no research that indicates a clear relationship between replicating the WCS and reducing the risk of injury [6].

The commonly analysed WCS may be of 1 min, 3 min, 5 min, or 10 min [23], but recreating the 1 min WCS during the session could serve to prepare our players for the maximum demands of competition [11]. There is a correlation between the WCS time and the conditional variable analysed (for example, variables relating to distances or accelerations, among others); the shorter the time analysed is, the higher is the WCS produced [2]. Therefore, the WCS is higher when analysing times of 1 min compared to 3 min, 5 min, or 10 min. Finally, these WCS could be influenced by contextual factors inherent to the match, such as the player’s position, the development of the match in the first or second half, playing the match at home or away, or being in a winning, drawing, or losing position during the match [2].

Due to the novelty of the present research topic, few studies have compared the WCS of training sessions with those of matches throughout an entire season in a professional team. Therefore, we considered it novel and interesting to conduct a detailed analysis of the conditional variables recorded throughout an entire season according to the positions of our players and compare them with the matches. This approach is novel because it not only examines weekly variations in WCS and provides practical information to optimise training. By identifying the specific WCS for each playing position during the week, coaches can ensure that training adequately prepares players to meet the demands of matches. In addition, this information enables targeted interventions, allowing coaches to identify the most appropriate days to train specific positional demands, thus improving both performance and recovery strategies.

Therefore, the main objective of this study was to analyse the worst-case scenarios (WCS) produced during the competition period throughout the week in various training sessions and to compare them with the physical demands of the matches. This comparison determines on which day of the week these maximum scenarios most closely resemble those experienced during a match over the season.

## MATERIALS AND METHODS

This longitudinal study was conducted on a professional football team over 40 weekly microcycles, excluding pre-season. The study included a total of 194 training sessions and 42 matches in the Spanish Second Division (Smartbank League) during the 2020/2021 season. Matches mainly occurred during uncongested competition periods, with one match per week. However, there were occasional matches during the congested competition periods, with two matches per week [24]. The training week was structured according to the competition schedule.

The training sessions were classified based on their proximity to the match day, as described in some previous studies [25]. The study analysed training sessions conducted on MD-5, MD-4, MD-3, MD-2, MD-1 (training held days before the match day) and MD (match day). Sessions conducted on MD-7, MD-6, and MD+1 were excluded from the study due to an insufficient and insignificant sample size.

Table 1 presents the general objectives and contents of each training session depending on the day it was conducted with respect to the MD.

**TABLE 1:** Goals and contents of the training sessions.

Session	Goals	Contents
MD-5	Physical	Strength
	Tactical	Small-sided games / Small-sided possession
	Technical	Ball control / Passing

MD-4	Physical	Endurance
	Tactical	Small-sided games / Large-sided possession
	Technical	Ball control / Shotting / Dribbling

MD-3	Physical	Strength / Endurance
	Tactical	Full-pitch match / Large-sided possession
	Technical	Ball control / Passing / Dribbling

MD-2	Physical	Speed
	Technical	Full-pitch match / Small-sided possession
	Tactical	Crossing & Finishing

MD-1	Physical	Neuromuscular
	Tactical	Small-sided games / Set pieces
	Technical	Shotting

MD	Physical	All
	Tactical	All
	Technical	All

MD-5 (training held five days before the match day), MD-4 (training held four days before the match day), MD-3 (training held three days before the match day), MD-2 (training held two days before the match day), MD-1 (training held one day before the match day) y MD (match day).

The Ethical Committee of Clinical Research of Aragón, Spain (CEICA), approved the present study under act nº 04/2021, with license PI21/060. The research was conducted in accordance with the Declaration of Helsinki. All participants were informed of the study objective and signed an informed consent form.

### Participants

Twenty-four male professional football players (age: 25.2 ± 4.5 years, height: 179.1 ± 5.9 cm, body mass: 75.0 ± 6.4 kg, body mass index (BMI): 23.3 ± 1.2 kg/m^2^) belonging to the Spanish Second Division (Liga Smartbank) were selected for the present study, as shown in Table 2. According to the Participant Classification Framework [26], the players are classified as belonging to the third level of competition, which is reserved for highly trained or national level athletes. However, for the analysis of each microcycle, only players who completed the full match MD match were included. Goalkeepers were excluded from the study because the physical demands of goalkeepers are totally different from those of outfield players. Participants trained for 9–10 hours per week (1.5–2 hours per day) and played one match during uncongested weeks. During the congested weeks, they trained for 7–8 hours per week (1.5–2 hours per day) and played two matches. The study analysed participants individually and by field demarcation, including central defenders (CD) (n = 5), wide players (WP) (n = 8), midfielders (MID) (n = 7), and forwards (FW) (n = 4). A total of 5053 observations were carried out, taking into account players, training sessions and, matches.

**TABLE 2:** Players by position

Position	n	Age (years)	Height (cm)	Weight (kg)	BMI
CD	5	27.6 ± 5.41	183.6 ± 4.34	77.20 ± 5.40	22.92 ± 1.72
WP	8	23.59 ± 1.55	176.29 ± 4.98	73.21 ± 5.61	23.53 ± 0.78
MID	7	26.25 ± 6.02	176.88 ± 5.25	73.00 ± 7.87	23.28 ± 1.55
FW	4	24.50 ± 5.07	185.50 ± 3.32	80.85 ± 2.22	23.47 ± 0.51

CD: Central defenders; WP: Wide Players; MID: Midfielders; FW: Forwards; BMI: Body Mass Index

### Instruments

The data for this study were obtained from GPS tracking devices, specifically the WIMU PROTM (RealTrack Systems S.L., Almeria, Spain), which have a 10 Hz GPS and a triaxial accelerometer with a frequency of 100 m0 Hz. The WIMU PROTM devices are considered valid and reliable for obtaining positioning metrics derived from GPS signals in football [27]. The software of this company was used to calculate the WCS. The devices were placed in a specially designed vest (Rasan, Valencia, Spain) with a pocket on the back for insertion. RealTrack Systems S.L. (Almeria, Spain), the owning company, calibrated the devices at the beginning of the season.

### Procedures

All sessions were conducted on the same natural grass training field. The athletes wore appropriate footwear for the surface and did not use shin guards. Sessions were held in the morning at the same time each day. The same warm-up routine was performed daily as an introduction to the main part of the session, which was tailored to the specific content being worked on. Throughout the season, the same coaching staff led all training sessions. During breaks between tasks, players were advised to drink water or isotonic drinks. A nutritionist supervised the diet (breakfast and lunch) and hydration of all players throughout the training weeks, ensuring optimal recovery for the athletes in preparation for the following sessions.

The software used for the analysis and processing of the data extracted from the selected variables was SPRO 960 (RealTrack Systems S.L., Almería, Spain). The variables chosen are as follows:

– Distance (metres) (DIST TOTAL): total distance travelled.
– Distance HSR (metres) (DIST 21): total distance run at a speed above the absolute high sprint running (HSR) threshold (default 21 km/h).
– Distance sprint (metres) (DIST 24): total distance run at a speed above the absolute sprint threshold (default 24 km/h).
– High accelerations (counts) (ACC): number of high-intensity accelerations (> 3 m/s^2^).
– High decelerations (counts) (DEC): number of high-intensity decelerations (< -3 m/s^2^).
– HMLD (metres) (HMLD): distance covered in high metabolic load actions (by default, above the threshold of 25.5 W · kg^−1^). Includes all high-speed running (speed greater than 21 km/h) and accelerations and decelerations exceeding 2 m/s^2^ [28].

Absolute thresholds were used for all variables in this study. This approach was chosen because although studies have suggested that relative thresholds may better individualise athlete workloads, the use of absolute thresholds ensures consistency and comparability across different athletes and studies [29]. Other studies have shown that the choice of analysing absolute or relative thresholds does not influence the planning and programming of training loads in athletes [30].

### Statistical analysis

The Kolmogorov-Smirnov test was used to confirm the normality of the data distribution and Levene’s test for equality of variances. A repeated measures analysis of variance (ANOVA) was used to identify differences in demand for training and match days. Bonferroni post-hoc analyses were then conducted when necessary to determine significant differences between training and match day for each playing position. Finally, effect sizes were calculated for all pairwise comparisons using Hedges’ *g*, with 95% confidence intervals. The interpretation of *g* was as follows: trivial = 0 to 0.19, small = 0.2 to 0.59, moderate = 0.6 to 1.19, large = 1.2 to 1.99, very large = 2.0 to 3.99, and near perfect ≥ 4.0 [31].

## RESULTS

**FIG. 1:**
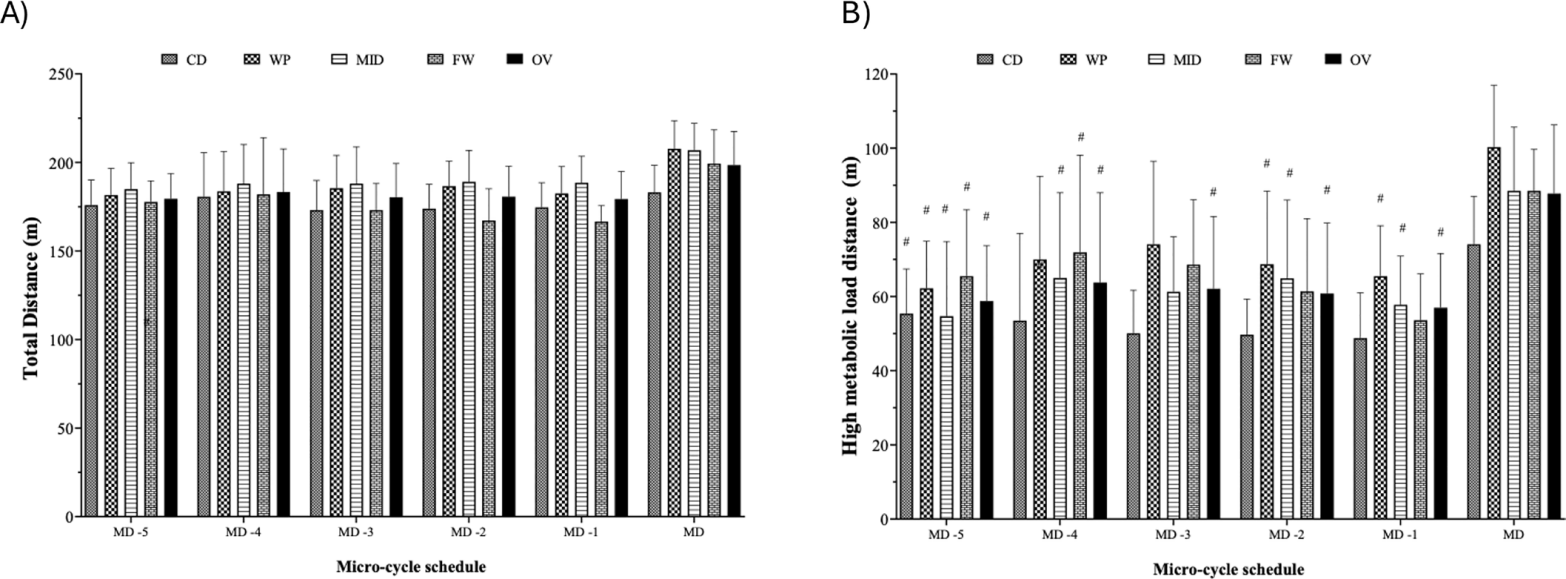
Micro-cycle patterns and comparative analyses of daily training workloads throughout a competition season, taking into account the field position and overall team in A) distance total and B) high metabolic load distance. #Significant differences with p < 0.05 between MD and training days. CD: Central defenders; WP: Wide players; MID: Midfielders; FW: Forwards; OV: overall; MD: match day; MD-5: five days before the match day; MD-4: four days before the match day; MD-3: three days before the match day; MD-2: two days before the match day; MD-1: one day before the match day

**FIG. 2:**
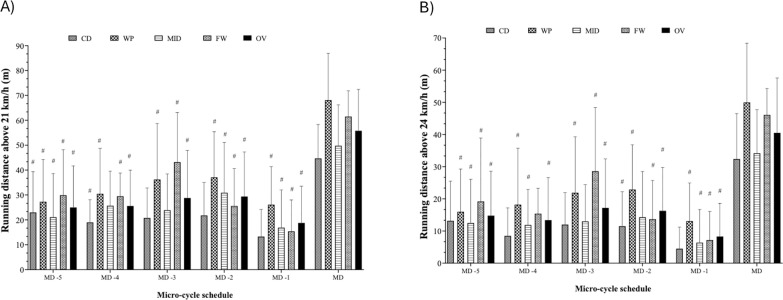
Micro-cycle patterns and comparative analyses of daily training workloads throughout a competition season, taking into account the field position and overall team in A) running distance above 21 km/h and B) 24 km/h. #Significant differences with p < 0.05 between MD and training days. CD: Central defenders; WP: Wide players; MID: Midfielders; FW: Forwards; OV: overall; MD: match day; MD-5: five days before the match day; MD-4: four days before the match day; MD-3: three days before the match day; MD-2: two days before the match day; MD-1: one day before the match day.

**FIG. 3:**
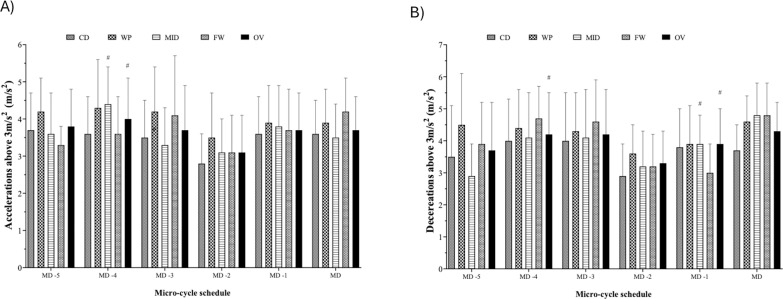
Micro-cycle patterns and comparative analyses of daily training workloads throughout a competition season, taking into account the field position and overall team in A) accelerations and B) decelerations exceeding 3 m/s^2^. #Significant differences with p < 0.05 between MD and training days. CD: Central defenders; WP: Wide players; MID: Midfielders; FW: Forwards; OV: overall; MD: match day; MD-5: five days before the match day; MD-4: four days before the match day; MD-3: three days before the match day; MD-2: two days before the match day; MD-1: one day before the match day

Figures 1, 2 and 3 display the micro-cycle and comparisons between every training day and the MD in terms of the total distance covered, high-velocity running (above 21 km/h and 24 km/h), accelerations and decelerations exceeding 3 m/s^2^, and high metabolic load distance considering the playing position. The repeated-measures ANOVA revealed no significant differences in the total distance covered within weekdays when comparing playing positions, as shown in Figure 1A. However, the total distance covered on MD represented 118.5% of the average training values (MD-5 to MD-1). High metabolic load distance was significantly lower on MD-1 compared to MD (p < 0.05), with no significant differences observed between MD and MD-4 for all playing positions (Figure 1B). The high metabolic load distance on MD represented 151.4% of the average training values.

When examining the differences between days, a significant difference was observed in running distance above 21 km/h and 24 km/h compared with MD across almost all playing positions (p < 0.05), except for CD, for which no significant differences were found when comparing MD with MD-3 and MD-1 in both variables (Figure 2). Notably, running distance above 21 km/h on MD represented 218.7% of the average training values, whereas the running distances above 24 km/h represented 289.3%.

Figure 3 highlights a significantly lower number of accelerations on MD-1 compared with MD for MID (p < 0.05). On the other hand, deceleration was significantly higher on MD-4 compared with MD for the same position (p < 0.05). Acceleration exceeding 3 m/s^2^ on MD represented 101.1% of the average training values. Deceleration exceeding -3 m/s^2^ on MD represented 111.4% of the average training values.

Table 3 presents the pairwise comparisons between each training day and MD in terms of total distance covered, high-velocity running (above 21 km/h and 24 km/h), accelerations and decelerations exceeding 3 m/s^2^, and high metabolic load distance. The results revealed no significant differences in total distance between pre-match days (MD-5 to MD-1) and MD (*p* > 0.05; ƞ*p*^2^ = 0.342). However, significant increases in the distance covered at speeds above 21 km/h and 24 km/h were observed on match days (*p* < 0.001; ƞ*p*^2^ = 0.854 and 882, respectively). Similarly, significant results were observed for accelerations and decelerations over 3 m/s^2^ compared to MD. In particular, accelerations on MD-4 demonstrated a significant difference (*p* = 0.006; ƞ*p*^2^ = 0.677). The high metabolic load distance also increased significantly on MD (*p* < 0.001; ƞ*p*^2^ = 0.899).

**TABLE 3:** Comparisons over competition season micro-cycles in terms of worst-case scenarios in Mean ± Standard Deviation.

	Microcycle period		Comparative	Mean difference (95%CI)	p	Hedge’s g (95% CI)
Total distance (m)	MD-5	138.9 ± 16.5	MD vs MD-5	11.3 (-26.4; 48.9)	0.295	-1.12 (-1.53; -0.71)
	MD-4	157.2 ± 13.8	MD vs MD-4	17.3 (-27.1; 61.6)	0.987	-1.06 (-1.50; -0.63)
	MD-3	159.4 ± 14.1	MD vs MD-3	33.1 (-28.5; 94.6)	0.999	-0.88 (-1.13; -0.62)
	MD-2	175.2 ± 9.05	MD vs MD-2	35.3 (-25.4;96.1)	0.965	-1.13 (-1.54; -0.72)
	MD-1	181.2 ± 3.03	MD vs MD-1	53.5 (-16.6; 123.7)	0.923	-1.40 (-1.83; -0.98)
	MD	192.5 ± 9.73

Running distance above 21 km/h (m)	MD-5	25.0 ± 16.7	MD vs MD-5	36.2 (22.6; 49.7)	< 0.001	-3.17 (-4.21; -2.13)
	MD-4	25.6 ± 14.4	MD vs MD-4	26.9 (16.9; 36.8)	< 0.001	-3.24 (-3.90; -2.58)
	MD-3	28.8 ± 19.1	MD vs MD-3	29.3 (15.4; 43.1)	< 0.001	-2.34 (-2.89; -1.80)
	MD-2	29.4 ± 17.9	MD vs MD-2	34.4 (22.1; 46.8)	< 0.001	-2.89 (-3.64; -2.02)
	MD-1	18.8 ± 14.7	MD vs MD-1	38.2 (22.7; 53.5)	< 0.001	-4.52 (-5.42; -3.62)
	MD	55.8 ± 18.8

Running distance above 24 km/h (m)	MD-5	14.8 ± 13.8	MD vs MD-5	29.3 (19.3; 39.4)	< 0.001	-2.30 (-3.00; -1.60)
	MD-4	13.4 ± 13.2	MD vs MD-4	23.4 (15.5; 31.3)	< 0.001	-3.54 (-4.27; -2.81)
	MD-3	17.2 ± 15.2	MD vs MD-3	22.4 (11.7; 33.1)	< 0.001	-2.01 (-2.47; -1.56)
	MD-2	16.3 ± 13.5	MD vs MD-2	27.8 (18.7; 36.9)	< 0.001	-3.37 (-4.30; -2.43)
	MD-1	8.3 ± 10.3	MD vs MD-1	28.7 (17.1; 40.4)	< 0.001	-4.05 (-4.93; -3.17)
	MD	40.5 ± 17.1

Accelerations above 3 m/s^2^ (m/s^2^)	MD-5	3.8 ± 1.0	MD vs MD-5	-0.03 (-0.65; 0.59)	0.986	0.22 (-0.44; 0.89)
	MD-4	4.0 ± 1.1	MD vs MD-4	0.82 (0.17; 1.45)	0.006	0.06 (-0.77; 0.89)
	MD-3	3.7 ± 1.2	MD vs MD-3	0.59 (-0.47; 1.66)	0.921	-0.10 (-0.44; 0.25)
	MD-2	3.1 ± 1.0	MD vs MD-2	0.45 (-0.77; 1.67)	0.953	-1.85 (-2.58; -1.13)
	MD-1	3.7 ± 1.0	MD vs MD-1	1.06 (-0.42; 2.53)	0.421	-0.12 (-0.83; 0.60)
	MD	3.7 ± 0.9

Decelerations above -3 m/s^2^ (m/s^2^)	MD-5	3.7 ± 1.5	MD vs MD-5	0.30 (-0.26; 0.85)	0.975	-1.20 (-2.25; .0.16)
	MD-4	4.2 ± 1.3	MD vs MD-4	1.28 (0.57; 1.98)	< 0.001	-0.05 (-0.62; 0.52)
	MD-3	4.2 ± 1.4	MD vs MD-3	0.69 (-0.68; 2.07)	0.923	-0.02 (-0.37; 0.32)
	MD-2	3.3 ± 1.0	MD vs MD-2	0.65 (-0.77; 2.08)	0.961	-1.78 (-2.28; -1.29)
	MD-1	3.9 ± 1.1	MD vs MD-1	1.78 (0.19; 3.37)	0.020	-0.37 (-0.84; 0.09)
	MD	4.3 ± 0.9

High metabolic load distance (m)	MD-5	58.8 ± 14.9	MD vs MD-5	30.7 (21.7; 39.6)	< 0.001	2.38 (-3.34; -1.42)
	MD-4	63.8 ± 24.2	MD vs MD-4	28.9 (14.2; 43.6)	< 0.001	-1.89 (-2.46; -1.32)
	MD-3	62.1 ± 19.5	MD vs MD-3	32.3 (9.02; 55.5)	0.002	-1.61 (-2.18; -1.04)
	MD-2	60.8 ± 19.0	MD vs MD-2	35.1 (13.3; 57.1)	< 0.001	-2.06 (-2.71; -1.40)
	MD-1	57.0 ± 14.6	MD vs MD-1	48.4 (21.5; 75.4)	< 0.001	-2.39 (-2.79; -1.99)
	MD	87.8 ± 18.5

MD-5, training held five days before the match day; MD-4, training held four days before the match day; MD-3, training held three days before the match day; MD-2, training held two days before the match day; MD-1, training held one day before the match day; MD, match day.

## DISCUSSION

The main aim of the study was to analyse the scenarios of maximum physical demand (WCS) produced during the week in the different training sessions and compare them with the physical demands of the match to determine which day of the week these maximum scenarios most closely resemble, in one season. Additionally, physical demands were compared between playing positions in relation to the training performed with respect to MD. There are studies suggesting that comparing training demands with match demands can help determine necessary modifications to training to meet match demands [3]. That is, understanding the demands of the match or training allows the selection of the most optimal training strategies to achieve the desired objectives within the microcycle.

The main findings were as follows: (i) MD showed the highest levels of training load compared to other days of the competition season, such as MD-5, MD-2 and MD-1. (ii) No significant differences were observed in the total distance covered in the different playing positions within the weekdays. (iii) Statistically significant greater distances covered above 21 km/h and 24 km/h were observed on MD compared to other days for most playing positions, except for central defenders (CD), among whom no significant differences were found on MD-3 and MD-1. (iv) A statistically significant lower number of accelerations was observed on MD-4 compared to MD for midfielders (MID), while a statistically significant greater number of decelerations was recorded on MD-1 for MID. (v) High metabolic load distance was statistically significantly lower on MD-1 compared to MD, while no significant differences were observed between MD and MD-4 across all playing positions. Therefore, pairwise comparisons revealed no significant differences in total distance between pre-match days and MD, but a notable increase in distances covered at higher speeds and accelerations/decelerations on MD, along with an increase in high metabolic load distance, indicating that match days are characterised by heightened physical demands and intensity, demonstrated by increased high-speed distances and metabolic loads.

An interesting study [32] analysed the physical demands by position in matches using variables similar to those used in the present study. In terms of total distance, the players with the highest WCS values are the midfielders (MID); in accelerations (> 3 m/s^2^) and decelerations (< -3 m/s^2^), they are the offensive midfielders (OMF); and in high metabolic load distance (HMLD), they are the MID. The data obtained in our study are not similar, as the players with the highest WCS values in total distance are the wide players (WP); in accelerations and decelerations greater than 3 m/s^2^, they are the forwards (FW); and in HMLD, they are the WP. These differences could be due to the different demands of each position when playing with one system of play or another [19].

In another, similar study [20], it was observed that on match days, players in wide positions (WP) had higher peaks in total distance, distance > 21 km/h, distance > 24 km/h, and HMLD compared to players in central positions. These data are in line with the values obtained in our study, in which the WPs had the highest peaks among the previously mentioned variables. Although only home matches were analysed in that study, the number of participants was similar, and the level of the athletes was equivalent to that in our research. Another recent study [16] showed that the players who reproduce the most WCS in total distance, high-intensity distance, or sprint distance on match day are the midfielders (MID). These studies do not match ours, because the players with the highest total distance and high-intensity distance are the WP. These differences could also be explained by the different demands of each position, depending on the playing system employed by each team [33].

Some studies have asserted that the dimensions of the tasks directly influence the physical demands obtained during the training week [34]. Based on their dimensions, these tasks can be classified as large sided games (LSG), medium sided games (MSG), or small sided games (SSG). An interesting study [35] showed that, during the week, the scenarios with the highest maximum demand correspond to training days that primarily include large sided games (LSG). Midfielders (MID) are the players who reach the highest peak of metres covered in one minute during training days and during the match. In all positions, the peaks reached during the week never exceed those reached on the match day. According to that study, the training days on which the highest WCS of the total distance covered should occur are MD-3 and MD-4. Our study found that the MID players had the highest WCS of total distance on MD-3 and MD-4. However, this was not the case on the match day, because the players with the greatest distance in the WCS were the WP instead of the MID. This difference could be due to the playing style used by the team during the season, in which the WP were important players in the team’s play, especially regarding play on the wings.

Previous research [36] indicated that the day of training with the highest volume and intensity relative to variables such as high-intensity metres covered or total distance would correspond to MD-3 in all positions. These findings are in line with our study regarding the high-intensity distance variable over 24 km/h, where the highest WCS values corresponded to MD-3 in nearly all positions. However, they do not align in total distance and distance over +21 km/h, as in our study, and the highest weekly values for total distance occurred on MD-4 and for distance over +21 km/h on MD-2. Nevertheless, other research [7] found that MD-4 and MD-5 were the days when the highest training demands in terms of volume and intensity typically occurred in the team, in all positions.

Also, in another study [37], the day of highest demand during the training week corresponded to MD-4, both in total distance and in the number of accelerations (> 3 m/s^2^) and decelerations (< -3 m/s^2^), which is in line with our study.

Not only are there studies analysing WCS in 1 min periods, but we can also find others [38, 39] analysing them in 5 min periods during competition. In the matches analysed in the first study [38], the players with the highest WCS peaks in acceleration, deceleration, and high-intensity distances were the WP. In the present study, based on 1 min durations, the highest WCS values for high-intensity distance variables were also observed in the WP. However, the FW exhibited higher acceleration and deceleration values. Both studies consistently indicated that players positioned on the wings tended to exhibit higher WCS values for total distance and high-intensity distances compared to those positioned centrally. Furthermore, in the second study [39], players who reproduced the most WCS in high-intensity distances in 5-minute periods were also the WP. Therefore, we can confirm that, generally, wide players require higher physical demands during competition.

When comparing the WCS produced in 1 min intervals with those produced in 3 min intervals, as demonstrated by some authors [40], divergent results were found. This study observed that MID players covered the greatest total distances during 3 min periods. However, our results, obtained by analysing WCS in 1 min intervals, showed that WP players covered the greatest distances. This discrepancy suggests that the player movement dynamics may vary significantly depending on the duration of the analysis interval. It is important to bear in mind that these differences may also reflect variations in the methodologies or contextual factors of the studies, such as differences in playing styles, tactical strategies, or physical conditioning across teams.

An interesting study [41] analysed similar conditional variables in the same demarcation as in our study in the WCS at 1 min, 3 min, 5 min, and 10 min in MD. Focusing on the WCS analysis of the 1 min period, we observed that the players who covered the most distance were the MID. In contrast, the players who covered the most distance at high intensity, accelerated and decelerated more times, and covered the most HMLD were the wing players. In our study, we found that, in 1 min periods, the players who covered the most total distance, the most distance at high intensity, accelerated and decelerated more times, and covered the most HMLD were the WP. Compared with our study, it only differed between the players who covered the most total distance during 1 min periods in the match. Without being able to precisely know the team’s playing model and tactical disposition in the study we are making a comparison to, these differences could arise because in the team we analysed in our study, the WP players had a significant role in the team’s gameplay development both in attack and defence. Therefore, comparing the results without knowing all the data raises a concern in this context.

Recently, a study [42] analysed the differences in WCS between the first and second halves of matches, without specifying whether only starting players, players who completed the entire match, or all players who participated in the match were included. In 1 min WCS, the players who covered the most distance in both the first and second halves were MID players. The players who had higher values of HMLD in both halves were also MID players. Although our study specifically analysed complete matches, the players who had higher values of total distance and HMLD were WP players. We cannot infer conclusions from this comparison because the difference between the analyses of the two halves individually and globally can be significant.

In recent years, research on the physical demands in women’s football has grown [43], allowing for comparisons with the physical demands of men’s football. When comparing the WCS without splitting into playing positions, the values found in the 1 min WCS were higher in men’s football for all variables. This difference is mainly attributed to the physical and morphological characteristics that distinguish men and women. [44].

Due to the novelty of the investigated topic, few studies in the scientific literature have compared WCS from training sessions with those from matches over an entire season in a professional team. Therefore, we considered it novel and interesting to conduct a detailed analysis of the conditioning variables recorded throughout a season based on our players’ positions and to compare them with match data.

However, we also identified a series of limitations in the development of this study. The first limitation was that MD+1 was not included in the study because players who completed the match, having performed recovery-oriented training, did not wear GPS devices. The second limitation was the decision to group both fullbacks and wide midfielders into a single group called ‘wide players’, since including only players who completed the entire match resulted in a very small sample size of wide midfielders throughout the season. Typically, substitutions in matches involve attacking players, which affects wide midfielders. The final limitation of the study was the sample size, as a professional football squad usually consists of 20–25 players distributed over several positions. Therefore, while analysing the data by playing positions can provide valuable information, the small sample size for each position limits the representativeness and generalisability of the results.

## CONCLUSIONS

It can be confirmed that, during the microcycle, high-demand scenarios typically occur during the MD. During the microcycle, no significant differences were found in any of the analysed playing positions in the total distance variable. However, significant differences were found in the variables of distance > 21 km/h and distance > 24 km/h in all positions except for the CD. In the MID position, MD-4 produced higher values than MD at acceleration > 3 m/s^2^. In addition, in decelerations < -3 m/s^2^, MD-1 produced higher values than MD. Finally, for the HMLD variable, MD-1 values were significantly lower compared to MD in all playing positions. Therefore, the reproduction of maximum demand scenarios during the week suggests a low injury rate. Future studies should examine the relationship between high-demand scenarios and injury rates or cumulative fatigue during the season. Investigating why physical demands vary between positions based on MD could help refine positional training strategies and improve recovery protocols. Finally, this study could help coaches or sport scientists to identify the most appropriate days to train specific positional demands, thus improving both performance and recovery strategies.
